# Supplementary material for: Are carbon emissions trading and green financial instruments synergistic? -Comprehensive quantitative research based on content analysis
Source: PLoS One. 2024 Mar 7;19(3):e0298601. doi: 10.1371/journal.pone.0298601 (PMC10919601; doi:10.1371/journal.pone.0298601)
Supplement: S1 Data — (ZIP) [file pone.0298601.s001.zip › Data/地级市Z_F工作报告.zip!/╣π╬≈/╣≡┴╓╩╨/└┤╫╘╣1⁄2╓┌║┼í╛┬φ┐╦╩2╛▌═°í┐_2011.docx]

各位代表：

现在，我代表市人民政府向大会作政府工作报告，请各位代表连同《桂林市国民经济和社会发展第十二个五年规划纲要(草案)》一并审议，并请各位政协委员和其他列席人员提出意见。

一、“十一五”和2010年工作回顾

过去的五年是不平凡的五年。面对国际金融危机的严重冲击，面对百年不遇的雨雪冰冻灾害，我们在自治区党委、政府和市委的正确领导下，科学研判、克难攻坚、开拓奋进，胜利完成了“十一五”规划确定的主要目标任务，预计2010年比2005年地区生产总值翻1.11番，人均地区生产总值翻1.06番，财政收入翻1.23番，工业增加值翻1.46番，规模以上工业企业利润总额翻1.82番，旅游总收入翻1.54番，全社会固定资产投资翻2.19番，社会消费品零售总额翻1.25番，进出口总额翻1.03番，实际利用外资翻2.30番，金融机构存、贷款余额分别翻1.37和1.40番；荣获“国家环境保护模范城市“全国绿化模范城市“全国双拥模范城“国家知识产权工作示范城市“全国人口和计划生育综合改革示范市“全国社会治安综合治理最高奖‘长安杯’“全国科技进步先进市“全国人民防空先进城市“全国创建文明城市工作先进城市“国家西部地区‘两基’攻坚先进地区”“中国国际友好城市交流合作奖”等荣誉。

——— 过去的五年，是综合经济实力跃上新台阶的五年。地区生产总值、财政收入实现千亿元和百亿元历史性跨越，地区生产总值由2005年的512.03亿元增加到2010年的1108.63亿元，年均增长13.5%；财政收入由51.61亿元增加到121.08亿元，年均增长18.6%。累计完成全社会固定资产投资2717.78亿元，年均增长35.5%。三次产业结构由23.4∶36.5∶40.1调整为18.3∶45.3∶36.4。以特色效益农业为主导的现代农业稳步发展，农林牧渔业总产值突破300亿元、增加值突破200亿元，农民人均纯收入突破5000元大关；粮食稳定增长，水果、蔬菜、食用菌种植面积和总产量居全区前列。以高新技术为先导的现代工业加速发展，工业主导地位显著增强，全市工业增加值由155.67亿元提高到427.61亿元，年均增长19.4%；完成技改投资529.62亿元，年均增长50.7%；食品饮料、机械电器产业超百亿元，新增工业上市企业3家、产值超亿元企业162家，工业对财政贡献率超过50%。以旅游业为龙头的现代服务业健康发展，质量效益显著提高，累计接待游客8601.90万人次，年均增长13.3%；实现旅游总收入549.74亿元，年均增长23.8%；社会消费品零售总额年均增长18.9%；金融机构存款余额由530.19亿元增加到1367.59亿元，年均增长20.9%。12县完成生产总值、财政收入、全社会固定资产投资占全市的比重分别由64.3%、41.8%、72%上升为67.8%、47%、78.5%，县域经济成为全市经济发展的重要力量。

——— 过去的五年，是基础设施建设实现新跨越的五年。“十一五”时期是我市历史上建设重大项目数量最多、规模最大、完成投资最多的时期，开工建设了一批事关桂林长远发展的重大项目。启动建设贵广高速铁路(桂林段)、湘桂铁路扩能改造工程(桂林段)；建设高速公路8条，通车总里程349公里；完成4E级标准机场跑道扩建工程；实现县县通二级以上公路、乡乡通油路目标；完成南洲大桥、穿山桥、雉山桥、东二环路、中隐路等一批城市路桥改扩建工程。永福电厂扩建等工程加快实施，完成电网改造投资31.75亿元，新增发电能力44.10亿千瓦时。完成153座病险水库除险加固，平乐巴江口水利枢纽设施投入使用，桂林市防洪及漓江补水枢纽工程顺利推进。

——— 过去的五年，是统筹城乡建设迈出新步伐的五年。深入实施“保护漓江，发展临桂，再造一个新桂林”发展战略，城镇化率达39%。临桂新区建设初见成效，完成规划编制，全面推进创业大厦、“一院两馆”[1]、中心公园、新区路网等一批重点项目。老城疏解提升成效显著，以“三桥十路”[2]和“1212”工程[3]为重点的基础设施项目加快推进；完成60公里城市道路2110栋临街楼宇立面整治改造。城区发展定位进一步明确，县城改造、新区建设进一步加快，特色鲜明的桂北城镇群初步形成。拓展深化城乡清洁工程和城乡风貌改造，城乡环境明显改善。扎实推进社会主义新农村建设，累计投入资金10.37亿元，是“十五”时期的4.70倍，惠及农户77.40万户297万人；完成20户以上自然村(屯)新农村规划编制1.06万个、新农村试点示范村建设354个；完成7021个自然村(屯)道路硬化工程，实现100%行政村通公路。累计投入资金13.52亿元，实施扶贫项目4894个，解决4.50万人温饱问题，8万人稳定脱贫，286个“整村推进”贫困村全部以优秀等次通过自治区验收。

——— 过去的五年，是生态文明建设取得新成效的五年。编制完成《桂林生态市建设规划》及12县《生态县建设规划》，并通过专家论证和市、县人大审议。全面启动“漓江四化”[4]“绿满八桂”造林绿化工程以及“两江四湖”二期综合整治工程，扎实推进科学保护漓江六大工程。全市森林覆盖率68.15%、城市绿化覆盖率44.3%，居全区前列；可建沼气池入户率　78.9%，持续位居全区第一；全面完成自治区下达的节能减排任务；市本级城市污水集中处理率90.4%，比“十五”期末提高41.8个百分点；城市生活垃圾无害化处理率100%；完成12县污水处理设施建设。环境质量指数在113个国家重点环境保护城市环境综合整治定量考核(“城考”)中名列前茅。被确定为全国首批循环农业示范市。12个乡镇创建“国家级生态乡镇”通过验收，阳朔镇获“国家级生态乡镇”称号。

——— 过去的五年，是推进改革开放增创新优势的五年。强力推进企业产权制度改革，完成国有和集体企业改革改制119户，搭建5个投融资平台，引进浦东发展银行等6家银行设立法人机构或分支机构，桂林市商业银行更名为桂林银行，启动新型农村金融改革试点。深化行政审批制度改革，减少投资审批事项100项。科教文卫、社会保障、农村税费、乡镇机构、土地流转、林权制度改革取得初步成效。全力推进“央企入桂”“百企入桂”，主动融入多区域开放合作，引进中国建筑股份有限公司、中国机械工业集团、中国中铁股份有限公司、大商集团、沃尔玛集团等一批国内外知名企业和世界500强企业落户桂林。累计新签市外投资项目3235个，项目总投资1693.05亿元，实际到位市外资金1276.92亿元，年均增长38%；新批外商投资企业150家，实际利用外资年均增长48.2%；进出口总额年均增长15.4%。

——— 过去的五年，是和谐社会建设取得新进展的五年。完成科研投入68.10亿元，是“十五”时期的1.66倍，获自治区级以上科技进步奖128项，创建自治区级以上企业技术中心30家、工程技术研究中心17家、国家地方共建联合实验室1家，获专利授权3045件。义务教育水平位居全区前列，职业教育攻坚任务全面完成，高考成绩名列全区前茅。打造并提升“百姓大舞台”“读书月”“百姓文化大讲坛”“漓江之声”和“印象·刘三姐”等一批文化品牌；在广西文艺创作铜鼓奖评比中继续位居前列；76%行政村建有农家书屋；“村村通”广播电视直播卫星覆盖工程全面完成。全民健身活动蓬勃开展。公共卫生体系不断完善。人口和计生工作保持全区领先水平，人口自然增长率控制在7‰以内。累计城镇新增就业31.69万人、国有企业下岗失业人员再就业8.47万人、农村劳动力转移新增就业41.10万人，城镇登记失业率控制在4.5%以内。城镇企业职工基本养老保险、失业保险覆盖率分别为100%、95.1%，城镇基本医疗保险覆盖率为92%，率先在广西全面建立城乡居民最低生活保障制度。坚持每年实施为民办实事工程，完成农村困难群众危房改造及“扶残安居”工程4131户，解决256万农民看病难、32.03万农村人口安全饮水问题，新建和改造农家店1243家。城镇居民人均可支配收入17949元、农民人均纯收入5487元，年均分别增长14.1%、12.8%，分别是2005年的1.93倍和1.83倍。

各位代表，刚刚过去的2010年，是“十一五”规划和科学发展三年计划实施的最后一年，经过全市人民共同努力，完成了市三届人大六次会议确定的主要目标任务。一年来，我们主要做了以下工作：

（一）强力推进“四大建设”，项目投资成效显著

全社会固定资产投资908.56亿元，增长37.8%，是“十五”时期的1.41倍。在建投资1000万元以上项目1796项，重大项目建设数量和投资规模创历史新高。

以临桂新区和老城基础设施“1212”工程为重点的城市建设完成投资200亿元，机场路等一批重大城市建设项目竣工使用，城市基础设施和公共服务设施进一步完善。交通水利基础设施建设完成投资120亿元，贵广高速铁路和湘桂铁路扩能改造工程征地搬迁基本完成，兴安至桂林高速公路超额完成年度投资计划，阳朔至鹿寨高速公路加快推进，灌阳至凤凰高速公路开工建设；小溶江、川江水利枢纽工程实现大江截流。工业园区完成固定资产投资90.80亿元，高新区完成土地平整1600亩，新建标准厂房2万平方米；苏桥经济开发区完成征地5617亩，新建标准厂房及服务楼3.71万平方米；秧塘产业园外扩4950亩，新建标准厂房5.50万平方米。以桂柳、桂梧、桂黄及桂阳公路沿线、漓江两岸和12县城为重点的城乡风貌改造完成投资43亿元，桂林市、阳朔县荣获全区城乡风貌改造优秀组织奖。

（二）加快工业结构调整，工业经济快速增长

完成工业总产值1263.44亿元，增长31.5%，其中规模工业总产值952.01亿元，增长37.1%；规模工业增加值310.26亿元，增长25.7%，工业经济运行质量和效益明显提高。

完成工业固定资产投资283.66亿元，增长29.4%。桂林福达集团比亚迪曲轴生产线等1343个项目开工建设；中橡桂林公司高等级子午线轮胎产业化等85个超亿元项目加快推进；桂林尚科光伏公司太阳能电池生产线(二期)等1158个项目竣工投产。完成技改投资216.05亿元，实施技改项目1589项。“五大五小”[5]工业产值724.69亿元，占规模工业总产值的76.1%。新增入园企业109家，园区工业增加值增长50.1%。建立市中小企业服务平台，整合组建中小企业信用担保公司，引进自治区金融投资集团，为50多家中小企业融资10多亿元，有力推动了中小企业发展。被列为广西“两化”融合[6]试点城市，荔浦县获“中国衣架之都”称号。

（三）提升特色效益农业，农村经济稳步发展

实现农林牧渔业总产值319.21亿元，增长5.1%；农业增加值202.60亿元，增长4.8%，湘江、资江、漓江、桂江四大流域和山区现代农业示范区建设初见成效。

深化调整区域、产业和品种结构，农业规模化、标准化、产业化和品牌化稳步发展。市级以上农业产业化龙头企业达119家、农民专业合作社达1590家，直接带动农户14.71万户。新增畜禽规模养殖场193个。引进农作物新品种703个，建立各种高产示范点252个，获得“绿色食品”标志产品45个，形成灵川小平乐村、临桂西版屋村等一批“一村一品”特色乡村，全州提子获全国中早熟优质葡萄评比金奖，平乐沙田柚获中国国际林博会金奖。实施农村公益事业项目1569个，新增农机总动力30.93万千瓦，完成冬春水利项目1527个，新增、恢复有效灌溉面积16.80万亩，改良农田94.43万亩。完成农民科技培训100万人次、农村劳动力转移培训9.50万人次。资源被评为中国果菜无公害十强县，临桂、灌阳获全国粮食生产先进县。

（四）推动旅游产业转型升级，第三产业态势良好

全社会消费品零售总额391.53亿元，增长18.9%。全年接待游客2246.33万人次，增长20.8%，其中入境游客148.62万人次，增长15.2%；实现旅游总收入168.30亿元，增长32.6%，旅游质量效益进一步提升。

编制完成桂林国家旅游综合改革试验区建设总体方案及规划纲要，成功争取桂林成为国家服务业综合改革试点区域。开展旅游服务质量提升年活动，加强旅游促销，拓展旅游合作，整治旅游秩序。愚自乐园二期、全州湘山寺景区等67个项目加快推进。新增4A景区7个、国家和自治区级农业旅游示范点4个。成功举办第四届联合国世界旅游组织/亚太旅游协会旅游趋势与展望国际论坛、首届中国桂林国际旅游博览会、桂林国际动漫节、“漓泉杯”2010第五届亚洲超级模特大赛颁奖礼，支持县(区)举办特色节事活动[7]，会展节事成为第三产业新亮点。阳朔被确定为首批全国旅游标准化试点县，秀峰区被评为全国养老服务示范单位。继续推进“家电下乡”“万村千乡”市场工程，“家电下乡”销售额增长148.3%，位居全区第二。全社会货运量增长25.3%。房地产投资118.01亿元，增长27.9%。深圳农商行、兴业银行、光大银行入驻开业；桂林国民村镇银行正式揭牌，成为全国首批地市级村镇银行；全市金融机构存、贷款余额分别增长23.2%和20%。

（五）强力推进城乡建设，城镇化发展步伐加快

完成城镇固定资产投资758.52亿元，增长39.1%，是“十五”期间投资总和的1.54倍，掀起了新一轮城市建设高潮。

修编完成《桂林市城市总体规划纲要(2010—2020)》并通过国家住建部批复，完成城北滨江区等15个片区16平方公里控制性详细规划。土地报批、规范使用、土地整理等工作位居全区前列。新区路网建设全面铺开，创业大厦、“一院两馆”等一批标志性项目相继开工，村民安置工作取得突破。金鸡路、建干北路快车道等一批道路完工通车，滨江北路等11条道路加快推进。“两江四湖”二期基本完成桃花江、小东江等河流清淤截污及生态河堤岸线建设工程；改造建设无障碍设施项目390多个，创建“全国无障碍建设城市”通过国家验收。完成城市房屋搬迁、违法建筑拆除100万平方米。迎宾路口、七里店路口等城市节点环境提升改造初显成效。訾洲公园建成开放，漓东公园开工建设，黑山植物园二期基础工作全面展开。秀峰区琴潭旅游文化休闲园路网构架初步形成，象山区旅游度假园已有多个项目进入，雁山区科教园中心环线一期、污水处理厂一期工程投入使用。12县完成县城主要道路硬化、绿化、亮化、美化工程，各县新区基础设施建设进度加快。

（六）加强节能减排和环境保护，生态文明建设卓有成效

实施桂林生态市建设规划，科学保护漓江取得明显成效，环境质量居全国重点城市前列，“城考”成绩继续位居全区第一。

编制《漓江风景名胜区总体规划》并上报国务院，推动自治区立法保护漓江，开展漓江支流(市区段)综合整治，完成造林1.88万公顷，山上造林和山下绿化完成率全区领先。耕地保护、卫片执法检查、地质灾害整治考核位居全区第一。提前完成自治区下达的年度淘汰落后产能任务，对18家水泥企业、54家锰业(铁合金)等耗能大户实行预警干预；规模以上工业万元增加值能耗下降11.04%，超额完成自治区指标6个百分点；新建14个城镇污水处理厂并投入运营。重点推进热电联产、风力发电、太阳能光伏、电动汽车等产业项目以及汽车加气站、充电站等设施建设，节能与环保产业快速发展。灌阳、恭城获国家首批绿色能源示范县。

（七）加快改革开放步伐，城市发展活力明显增强

政府机构改革有序推进，“三定”方案基本落实。市本级和临桂、兴安、永福、恭城等县级基层医疗卫生机构综合改革试点全面启动。集体林权制度改革任务基本完成，年度林地勘界面积2190.19万亩，发证面积1546.94万亩。深化国有、集体企业和供销体制改革，引进香港溢达、南方建材等一批战略投资者并购重组我市企业。主动融入广西“两区一带”，推进磨盘山客运港、平乐印山和阳朔旅游码头工程。开辟桂林至新加坡、银川经桂林至三亚、临沂经桂林至海口3条航线，恢复桂林至曼谷航线，增加桂林至台湾航班。圆满完成赞比亚、加蓬两国总统等国外贵宾友好访问接待任务，与波兰托伦市结为友好城市。招商引资成效显著，新签市外境内项目502个，总投资386.46亿元，增长17.9%，排名全区第二；实施市外境内项目899个，实际到位资金374.13亿元，增长33.2%；实际利用外资2.5亿美元，增长25.3%，排名全区第三。进出口总额9.03亿美元，增长22.6%。临桂被评为全国最具投资潜力中小城市百强县。

（八）全力保障和改善民生，和谐社会建设扎实推进

加强就业和社会保障工作。免费培训下岗失业人员1.38万人次，提供公益岗位4856个，新增就业6.89万人，城镇登记失业率3.95%。5项社会保险[8]参保人数230.08万人次，社保基金征缴32.71亿元，创历史新高；兴安县新型农村养老保险制度试点全面实施，参保率87.3%；按自治区标准筹发补助城乡低保对象38.99万人；改造农村危房8550户，解决救灾专项资金3066万元；经济适用房竣工26.99万平方米。

扎实推进惠民工程。继续从解决群众最关心、最直接、最紧迫的问题入手，筹资18.73亿元，全面完成教育惠民、医疗卫生保障、社会保障、文化惠农、安居惠民、农村基础设施建设、城乡社会服务、蔬菜安全检测、城市水环境和交通环境综合整治等10项惠民工程[9]。

社会事业全面进步。扎实推进第4轮创新计划和科技“355工程”[10]，全社会科研投入15.80亿元，实施技术创新项目562个，完成专利申请1030件，专利授权813件，居全区前列。发放各类助学金8060.95万元，惠及高中阶段学生24.82万人次；完成125个中小学校舍安全工程项目，完成培智学校迁建工作，聋哑、七中两校迁建和市职教中心临桂分校建设取得阶段性成效。“漓江之声”获第十五届全国群星奖项目奖，《偷秋》获第四届全国少数民族曲艺展演节目一等奖，“百姓大舞台”等各类群众文化演出1.28万场；成功举办第二届中国山水画艺术双年展、2010中国桂林·史前文化遗产国际高峰论坛；靖江王府及王陵、甑皮岩遗址入选第一批国家考古遗址公园立项名单，新增非物质文化遗产传承基地5家；建设村级公共服务中心58个、乡镇综合文化站32个、农家书屋386家、三级文化信息资源共享工程1021个。实施医疗卫生服务体系建设项目106个，完成农村改厕1.55万座，创建文明卫生村400个，通过“国家卫生城市”复审。全民健身活动蓬勃开展，完成农村体育健身工程示范点153个；我市运动员马欢欢获得亚运会女子水球团体金牌。我市“诚信计生”经验在全国推广，市计生协会获全国地级协会工作先进单位，叠彩区获“全国计划生育优质服务先进县(区)”。市老龄办获“全国老龄工作先进单位”。《桂林年鉴(2009)》获全国地方志系统第二届年鉴奖一等奖，市志办获“全国方志系统先进集体”。成功举办恭城瑶族自治县成立20周年庆典活动。积极推进社会矛盾化解、社会管理创新、公正廉洁执法工作，社会综合防控体系进一步完善，刑事案件侦破率和治安案件查处率显著提高，开展大接访、大排查、大调解、大防控活动，化解各类矛盾6万多起，突发性、群体性事件得到依法妥善处置。“五五”普法成效显著，通过“全国普法先进城市”验收。食品药品安全和安全生产形势总体稳定。

国防建设、国防动员、民兵预备役、人民防空及双拥工作得到加强，宗教、人事、审计、统计、编制、接待、机关事务、档案、供销、防震减灾、侨务、发展研究、社会科学、妇女儿童、残疾人等事业取得新进展，中直、区直驻桂单位取得新成绩。

一年来，我们坚持科学决策、民主决策，坚持重大事项向市人大及其常委会报告、向市政协通报制度，办理市人大代表建议、批评和意见98件，市政协提案285件，办结率100%，被自治区政协评为先进承办单位。政府廉政建设工作得到加强。坚持广泛听取各民主党派、工商联、无党派人士意见，支持工会、共青团、妇联等群团组织发挥作用，凝聚各方智慧，共同推动我市经济社会平稳较快发展。

各位代表，过去的五年，是磨砺艰辛的五年，是成就显著的五年。通过五年努力，我市工业化进入产业升级、结构优化、做大做强的新阶段，城镇化进入快速成长与质量提升并重的新阶段，农业发展进入特色化、规模化、品牌化的新阶段，服务业进入改革创新、加快发展的新阶段，区位优势、政策优势、人才优势、金融优势日益凸显，百里漓江春潮涌动，桂北大地活力迸发，桂林已经站在一个新的历史起点上！

回顾五年的工作，我们的体会是：必须坚持深入学习实践科学发展观，加快转变经济发展方式，推动经济又好又快发展。必须坚持解放思想，改革创新，破除发展体制机制障碍，增强发展动力与活力。必须坚持调整优化经济结构，增强自主创新能力，统筹城乡发展，提升发展质量和效益。必须坚持深化开放合作，以大开放促进大合作，以大合作促进大发展。必须坚持加强生态文明建设，加强节能减排，切实保护和改善环境，促进人与自然协调发展。必须坚持以人为本，切实保障和改善民生，让人民群众共享改革发展成果。必须坚持求真务实作风，出实招、办实事、求实效，埋头苦干、团结拼搏，不断创造发展新优势。

各位代表，五年成就来之不易。这些成绩的取得，是自治区党委、政府和市委正确领导的结果，是市人大、市政协监督支持的结果，是全市人民齐心协力、奋发进取和各方面关心帮助的结果。在此，我代表市人民政府，向全市各族人民，向给予政府工作大力支持的人大代表和政协委员，向各民主党派、工商联和社会各界人士，向中央、自治区驻桂单位，向驻桂人民解放军、武警部队官兵，向所有关心、支持桂林发展的海内外朋友，表示崇高的敬意和衷心的感谢！

回顾五年工作，我们清醒地认识到，我市经济社会发展仍然存在一些不容忽视的问题。主要表现在：我市仍属后发展欠发达地区，经济总量较小，经济结构不够合理，产业竞争力不够强；城镇化水平偏低，区域发展不平衡；节能减排压力增大，土地和环境约束日益突出；体制机制障碍仍然影响发展，社会转型和利益调整引发的社会矛盾依然存在，创业发展环境还需改善，等等。对此，我们要高度重视，在今后工作中采取更有力的措施加以解决。

二、“十二五”发展目标和主要任务

今后五年，是全面建设小康社会的关键时期。经济全球化进程加快，国内经济结构转型升级，西部大开发战略继续实施，中国―东盟自由贸易区全面建成，国务院《关于进一步促进广西经济社会发展的若干意见》贯彻落实，桂林国家旅游综合改革试验区和服务业综合改革试点区域建设全面推进，我市迎来千载难逢的历史机遇。同时，也面临诸多不利因素和巨大挑战。我们必须增强机遇意识、责任意识、忧患意识，再接再厉、扎实工作，在更高起点上勇攀新高峰！

根据市委三届十次全会精神，“十二五”期间我市经济社会发展的指导思想是：以邓小平理论和“三个代表”重要思想为指导，全面贯彻党的十七大、十七届五中全会和自治区党委九届十三次全会精神，深入贯彻落实科学发展观，围绕“富民强桂”新跨越的目标要求，坚持以科学发展为主题，以加快转变经济发展方式为主线，深入实施西部大开发战略，深化改革开放，坚持走农业稳市、文化立市、旅游兴市、工业强市之路，全力实施“保护漓江，发展临桂，再造一个新桂林”发展战略，全面推进桂林国家旅游综合改革试验区和国家服务业综合改革试点区域建设，保持经济平稳较快发展和社会和谐稳定，保障和改善民生，不断开创建设现代化国际旅游名城、历史文化名城、生态山水名城新局面，为全面建成小康社会打下具有决定性意义的基础。

我市“十二五”规划指导思想的一个鲜明特点，就是围绕自治区“富民强桂”新跨越的目标要求，明确提出了坚持农业稳市、文化立市、旅游兴市、工业强市的发展路子和建设现代化国际旅游名城、历史文化名城、生态山水名城的战略定位。这是基于我市经济社会发展现状、资源禀赋和产业发展要求以及面临的重大发展机遇而提出的，其根本目的就是实现“富民强市”。“富民”就是更加注重以人为本、富民优先，更加注重统筹城乡区域发展，更加注重保障和改善民生，更加注重促进社会公平正义，不断满足人民群众过上更好生活的新期待；“强市”就是聚精会神搞建设，一心一意谋发展，做大做强特色优势产业，全面增强综合实力、整体竞争力和可持续发展能力。

《纲要(草案)》提出了7项基本要求：坚持转变方式，努力实现科学发展；坚持改革开放，努力实现创新发展；坚持发挥优势，努力实现带动发展；坚持重点突破，努力实现优先发展；坚持统筹兼顾，努力实现协调发展；坚持生态建设，努力实现绿色发展；坚持民生为本，努力实现共享发展。这7项基本要求，是促进我市科学发展的重要体现，是未来5年我们努力的方向和工作重点。

《纲要(草案)》提出了“十二五”时期经济社会发展的目标：经济平稳较快发展，地区生产总值年均增长11%，力争到2015年实现地区生产总值比2010年翻一番，财政收入、全社会固定资产投资、社会消费品零售总额、进出口总额翻一番以上；经济结构调整取得重大进展，三次产业结构调整为13∶48∶39，城镇化率达50%；九年义务教育巩固率提高到93%，科技教育发展明显加快，研发经费支出比重明显增加；生态文明建设取得明显成效，生态环境质量保持全国领先水平；人民生活全面改善，城镇居民人均可支配收入和农民人均纯收入与经济发展同步增长；社会建设明显加强，保障体系更加完善，服务体系更加健全，社会更加和谐稳定；重点领域和关键环节改革取得明显进展，非公经济占国民经济的比重大幅提高，开放合作水平进一步提升。

《纲要(草案)》明确了“十二五”期间打造“一城二区三中心四基地”[11]的战略任务，提出了经济发展、科技教育、资源环境、人民生活4大类共35项具体指标，其中预期性指标19项、约束性指标16项。这些指标与以往相比，指标数量减少，重点更加突出，目标更加清晰，体现了科学发展的要求，体现了加快转变经济发展方式的要求，体现了到2020年实现全面建设小康社会奋斗目标的要求。

实现上述目标任务，需要把握以下战略重点：

（一）更加注重经济结构调整，加快转变发展方式

构建以特色效益农业为主导的现代农业体系。深入实施“农业稳市”战略，围绕“富民、强县、奔小康”目标，全面建设规模农业、品牌农业、设施农业、生态农业、休闲农业，大力拓展农业经济功能、生态功能和服务功能，推动农业产业结构优化升级，发展壮大县域经济，建设社会主义新农村，实现由农业大市向农业强市转变。

构建以高新技术产业为先导的现代工业体系。深入实施“工业强市”战略，坚持把工业化、信息化作为加快转变发展方式的主导方向和核心战略，以信息化推动工业化，促进产业结构优化升级，发展壮大优势产业，培育战略性新兴产业，加快发展园区经济，形成以城区高新技术产业中心为核心、以临苏经济产业带为腹地、以各县工业集中区为节点的工业发展新格局。

构建以旅游业为龙头的现代服务业体系。深入实施“旅游兴市”战略，全面建设桂林国家旅游综合改革试验区，实现旅游业发展由观光型向多元复合型、粗放型向集约型、规模数量型向规模质量效益型转变，打造国际一流旅游目的地和游客集散地。全面建设桂林国家服务业综合改革试点区域，建立规范有序的服务市场体系，重点发展生态旅游、商贸物流、商务会展、保健养老、文化创意、金融保险等服务业，打造区域性商贸中心城市、会展中心城市、养老服务业基地，走出三次产业协调发展的新路子。

（二）更加注重基础设施建设，提高发展支撑能力

着力构建公路、铁路、民航等综合交通运输体系，建设区域性综合交通运输枢纽，打造桂粤湘黔交界区域重要中心城市。统筹推进能源水利基础设施建设，构建开放、多元、清洁、安全、经济的能源保障体系。加快推进信息基础设施建设，提高城乡信息化发展水平。加快完善城乡公共服务设施，提高城乡公共服务能力。

（三）更加注重城乡统筹发展，加快推进城镇化

按照自治区要求，力争构建中心城市人口规模120万的特大城市，优化提升老城区，基本建成临桂新区，全面建设苏桥产业新城；按照中等城市目标规划建设阳朔、临桂、灵川、全州、兴安、永福、平乐、荔浦等县城；按照小城市目标规划建设灌阳、龙胜、资源、恭城等县城；规划建设一批特色乡镇，形成以中心城市为核心，中小城市、特色乡镇协调发展的桂北城镇群。

（四）更加注重推进改革开放，全面提升城市软实力

深入实施“文化立市”战略，树立人才是第一资源理念，优先发展教育，加强文化创新，完善科技创新体制机制，促进文化大发展大繁荣。继续推进重点领域改革，转变政府职能，提高行政效能。加强国内外交流合作，扎实推进“央企入桂”“名企入桂”，为推动科学发展、跨越发展、和谐发展提供制度保障和动力源泉。

（五）更加注重生态环境保护，建设生态文明城市

全面实施生态市建设规划，强化以漓江为重点的生态环境保护，加强污染综合治理，完善水资源和水环境保障体系以及防灾减灾体系。强力推进节能减排，强化资源节约与利用，培育以绿色经济为特征的经济体系，加快形成节约能源资源、保护生态环境和有利于改善气候环境的产业结构、增长方式和消费模式，构建资源节约型和环境友好型社会。

（六）更加注重保障和改善民生，促进和谐社会建设

建立健全基本公共服务体系，推进城乡基本公共服务均等化。实施更加积极的就业政策，稳定就业形势。合理调整收入分配，提高居民收入在国民收入分配中的比重。建设覆盖城乡的社会保障体系和基本医疗卫生服务体系。完善社会救助机制，提高灾害救助水平。坚持计划生育基本国策，稳定低生育水平。实施全民健身工程，提高竞技体育成绩。加强社会主义精神文明建设和民主法制建设，推进社会管理创新，完善社会治安防控体系，确保社会和谐稳定。

三、2011年主要工作部署

2011年是中国共产党成立90周年，也是实施“十二五”规划开局之年，开好头、起好步至关重要。根据我市经济社会形势分析、发展条件和“十二五”规划目标，按照市委“一推两改”[12]的总体工作思路，今年全市经济社会发展的主要预期目标是：生产总值增长11%，财政收入增长13%，全社会固定资产投资增长20%，社会消费品零售总额增长16%，外贸进出口总额增长15%，城镇居民人均可支配收入、农民人均纯收入分别增长10%，城镇登记失业率控制在4.5%以内，人口自然增长率控制在8‰以内，居民消费价格涨幅控制在5%左右。实现上述目标，要重点抓好以下十个方面工作：

（一）突出抓好“四大建设”，增强经济发展新动力

继续开展以城市建设、交通基础设施建设、园区建设、城乡风貌建设为重点的“项目建设年”活动，全年计划完成全社会固定资产投资1090亿元，实施第一批重点项目643项，计划投资458亿元。

强力推进“四大建设”。继续推进以临桂新区、老城改造、各县新区建设为重点的城市基础设施和公共服务设施建设，力争完成城市建设投资250亿元。强力推进以“两铁八高三库”[13]为重点的基础设施项目建设，力争完成交通、水利、能源等基础设施建设投资150亿元，重点推进贵广高速铁路、湘桂铁路扩能改造，协调推进桂林两江国际机场航站楼建设，实现兴安至桂林高速公路建成通车，加快推进灌阳永安关至全州凤凰、阳朔至鹿寨等高速公路和干线公路网、农村公路通畅工程，开工建设桂林至三江高速公路，加快资源至兴安、桂林至柳城、灌阳经恭城至平乐、兴安至龙胜高速公路、苏桥至永福一级公路及桂林北客运枢纽工程[14]、桂林西货运枢纽工程[15]等项目前期工作；统筹推进国电永福发电有限公司2×350兆瓦上大压小热电联产扩建工程等能源项目建设；全力实施桂林市防洪及漓江补水枢纽工程。继续推进工业园区基础设施建设和项目入园工作，力争完成园区建设固定资产投资100亿元。继续推进城乡风貌改造向纵深发展，重点实施一批城市街区、节点、旅游通道、城镇风貌改造项目，力争完成城乡风貌建设投资50亿元。

强化财政金融支撑。加强财政金融工作，大力培植财源，优化支出结构，加大财政对重点项目的支持力度。加快整合、盘活政府资源，做强做大做优政府投融资平台。加强项目审计工作，确保项目工程质量和资金安全。完善政府协调管理金融的体制机制，改善金融生态环境。继续实施“引金入桂”，不断完善金融组织体系。综合运用BT、BOT等方式，努力扩大项目融资规模。继续支持有条件的企业直接融资，争取1至2家企业上市、发行企业债券10亿元以上。积极引导各类资金投向旅游业、现代服务业、高新技术产业及交通能源、农林水利、社会民生等重点领域。

强化项目工作保障。策划和储备一批转方式、调结构、惠民生的重大项目，争取更多项目进入国家和自治区投资计划。做好以前期经费、审批程序、服务体系为重点的保障工作，下放项目审批权限，减少项目审批环节，推进部门联合审批，扎实开展“工作落实年”活动，改进机关作风，完善项目管理责任制、目标考核责任制，强化项目跟踪检查、协调服务和监督管理机制，着力解决制约项目推进的用地、环评、资金、搬迁、安置等问题，促进项目尽快开工、竣工达产。

（二）突出抓好“两大改革”，争创经济发展新优势

探索现代服务新模式，丰富现代旅游新内涵，打造国际一流旅游目的地和游客集散地，发展一批旅游强县和特色旅游城镇，接待游客总人数增长9.4%，旅游总收入增长19%。

强力推进桂林国家旅游综合改革试验区建设。争取国家、自治区批复《建设桂林国家旅游综合改革试验区总体方案》，积极争取政策支持，力争在旅游政策、旅游规划、旅游行政管理体制改革、旅游综合执法体制改革和漓江经营管理模式改革等方面取得突破。整合旅游资源，完善中心城区集散功能，发展阳朔、兴安两个旅游增长极，推进桂林—阳朔—荔浦—平乐—恭城、桂林—资源—龙胜、桂林—灵川—兴安—全州—灌阳、桂林—临桂—永福等4条发展轴带建设，健全景区景点交通干线网络，打造1小时旅游圈。培育三大特色产品，开发三大水陆游线，形成七大旅游品牌[16]。打造一批旅游精品，重点推进靖江王府及王陵大遗址和甑皮岩国家考古遗址公园、桂林飞虎队遗址公园、阳朔十里画廊景区、龙胜龙脊景区、兴安灵渠景区、资源天门山景区、灌阳千家洞景区、全州天湖景区等项目建设。深化旅游合作，开辟国际新航线，拓宽客源新市场，加快游客集散中心建设，建立网络化旅游服务系统。办好第五届联合国世界旅游组织/亚太旅游协会旅游趋势与展望国际论坛、第二届中国桂林国际旅游博览会、第三届桂林国际山水文化旅游节、桂林国际动漫节等节事活动。

全面启动国家服务业综合改革试点区域建设。积极争取国家和自治区政策支持，建立健全服务业发展组织机构和工作机构，完善服务业发展各类专项规划，出台相关政策措施。大力推进服务业与一、二产业融合发展。培育发展文化创意、社会化养老等新兴服务业，启动建设国家级服务外包基地城市，做好全国云计算服务[17]试点城市申报前期工作；建设养生度假和社会化养老产业发展示范区，重点推进康复养生养老基地建设。大力发展商贸物流、商务会展等生产性服务业，建设商务会展产业与旅游产业融合发展示范区，重点推进以七星区为核心的旅游与会展资源整合及产业协同项目；建设以老城区和临桂新区为“双核”的商贸物流聚集示范区，重点推进航空港保税物流园区等区域性商贸物流项目规划立项，加快建设一批区域性批发市场。积极发展生态旅游、休闲娱乐等生活性服务业，建设乡村生态休闲旅游度假示范区，重点推进以阳朔为基地的城乡互动乡村生态休闲旅游示范工程、以雁山为基地的观光休闲旅游带；建设体育文化特色消费示范区，重点推进叠彩城北滨江区以站前广场为节点、漓江两岸为轴带的文化、休闲、体育项目建设。继续实施“家电下乡”、“建材下乡”、“汽车下乡”及农超对接，完善城乡商贸流通体系，重点抓好农家店和配送中心建设。

（三）以转变发展方式为主线，实现工业经济新突破

以高新技术产业为先导，发展园区经济，调整产业结构，完善产业体系，实现规模工业总产值增长23%、增加值增长17%，技术改造投资增长25%。

加快发展高新技术产业。继续发挥桂林国家高新技术产业开发区的引领、示范、辐射、带动作用，加快提升创新能力和孵化能力，大力发展电子信息、生物医药、高端装备制造业；加快制定规划，培育发展新材料、新能源及新能源汽车、节能与环保等战略性新兴产业，力争实现高新技术产业产值520亿元，增长30%。重点推进中国电子信息集团桂林电子配件基地(苏桥)、中国机械工业集团桂林电器研究院电工电子新材料产业化基地、桂林福达集团年产10万吨精密锻造中心(二期)、兴安光伏产业园、桂林尚科光伏公司太阳能电池生产线技术改造项目、桂林鑫友光伏公司太阳能电池组件项目、桂林众阳光能公司光伏电池项目、资源金紫山风能发电(二期)、龙胜南山风电场、桂林啄木鸟医疗器械生产基地、桂林海威科技LED生产线扩建(二期)等项目建设。

提升工业整体素质。实施工业化和信息化融合示范工程及试点工作，重点促进机械装备、轻工食品等行业信息化建设。积极运用先进适用技术和信息技术改造提升传统产业，力争完成亿元以上技改项目30个。围绕电子信息、汽车、机械、食品、生物医药，打造百亿元产业；支持优势企业，鼓励配套企业，力争产值超亿元企业250家、10亿元企业15家，重点推进苏桥新能源客车及产业链配套生产基地、中橡桂林公司高等级子午线轮胎产业化、桂林娃哈哈食品有限公司饮料生产线扩建(三期)、桂林南药股份公司青蒿琥酯高技术国际化产业、荔浦桂林微邦生物技术有限公司魔芋甘露低聚糖产业化、桂林莱茵生物科技股份有限公司标准化植物提取物加工产业化工程(二期)等78个超亿元项目建设进度，力争30个以上重点项目竣工投产。

加强工业园区建设。把园区建设与新区建设结合起来，按照规划先行、配套完善、项目跟进、集聚发展的思路，推动扩区工作，打造国家高新技术产业开发区、西城经济开发区等百亿元工业园区。提高园区产业配套、物流配送和商务生活等综合服务能力，完成园区基础设施建设投入15亿元，增长25%以上，加快形成客车产业园、电工电气产业园、荔浦衣架产业园等一批特色园区。发挥苏桥经济开发区、八里街开发区和各县工业集中区等各类园区作用，优化产业布局，提高园区企业投资强度、单位土地产出率和园区土地集约利用水平。新增入园企业(项目)100个以上，工业园区主要经济指标增长明显高于全市平均水平。

加大服务企业力度。继续深入开展“企业服务年”和市长“企业接待日”活动，加大对工业发展和园区建设的资金支持力度，做大市中小企业信用担保公司，组建中小企业担保协会，拓宽融资渠道，提高企业融资规模和比重，切实解决中小企业融资难问题。深入实施中小企业成长工程，完善公共服务平台，建立覆盖全市的中小企业社会化服务网络。

（四）以现代农业为主导，促进农村经济新发展

发展特色效益农业，完善公共服务，推动农业发展与新农村建设、旅游业、城镇化相结合，实现农林牧渔业增加值增长4%。

加快特色效益农业发展。深入实施“南提北扩”战略和湘江、资江、漓江、桂江及山区现代农业发展规划，创建特色效益农业、乡村旅游农业、吨粮万元田、循环农业示范市。确保粮食播种面积560万亩，推进新一轮“菜篮子”工程，巩固生猪、家禽产业，做大做强水果、食用菌、马铃薯、中药材产业，重点打造阳朔金桔、恭城月柿、资江红提、尧山花卉等一批产业基地。在稻作区推广稻—灯—鱼—菇、稻—菜—薯，在园艺作物区推广套种马铃薯、蔬菜等高效农业模式。实施水肥一体化技术，加快区域物流冷链中心建设。调整林种结构，培育市区竹木加工贸易，灵川临桂永福人造板，兴安竹制品加工等产业。改造提升一批乡村农业旅游示范点，推进城乡产业联动发展。

加强农村公共服务建设。有效整合各项涉农资金，加快主导农产品地方标准、动植物疫病防控和保护、农产品质量安全检验检测、农机推广服务等公共服务体系建设。大力发展农业生产经营服务组织和农民专业合作组织。抓好市县农业科研单位体制创新，加快广西农科院桂北分院、瑞克思旺(桂林)育种站、中国农科院南方重大病虫野外观察站等内引外联科研机构建设。继续开展百万农民科技大培训，努力实现百万农民转移就业。

推进社会主义新农村建设。加强农村水、电、路、气、通信等基础设施配套建设。加快中低产田改造，建设旱涝保收高标准农田。完成30座水库除险加固，实施乡村污水垃圾处理工程。进一步强化乡村文化体育设施建设、乡村风貌环境卫生综合整治，加快普惠制新农村建设。抓好第三批82个贫困村“整村推进”工作。

（五）以中心城市为主战场，塑造城乡建设新风貌

加快建设环境优美、山水魅力、历史文化、和谐宜居的特大城市步伐，城镇化率提升2.2个百分点。

全面加快新区建设。进一步完善临桂新区功能规划，力争行政区划调整取得实质性进展。按照“一主三辅两组团”[18]的思路，推动临桂新区建成新的政治、经济、旅游、文化中心，形成大旅游、大产业、大物流的新城区。实施项目54项，年度投资32.6亿元。创业大厦基本建成，“一院两馆”部分场馆投入使用，中心公园项目初具规模，“五纵三横”[19]新区路网、农民安置新村、水利防洪排涝、湖塘水系及核心区给水、排水、电信、电力等工程基本建成。加快推动金融大厦、广播电影电视中心、报业传媒中心、新闻出版图书发行大厦、交通运输枢纽指挥中心、市民广场等一批带动性项目建设；加快推动临苏路、临雁路[20]、凤凰西路、临桂新区城市公共交通及公交枢纽等交通设施项目建设；加快推动新区大水系公园、体育运动休闲公园、旅游会展、旅游论坛、科技馆、文化宫、咨询服务中心等公共服务项目建设。加快推进会仙湿地公园景区保护、规划、建设、开发和万福休闲旅游景区规划立项工作，形成旅游产业与新区互动发展；加快推进综合物流园区、现代物流配送中心规划立项建设，形成物流园区与新区互动发展；加快推进秧塘产业园、苏桥产业园和临苏产业带建设，形成产业园区与新区互动发展。扎实推进各县新区建设。

切实疏解提升老城。继续推进市容提升计划[21]，完善市域综合交通规划。在继续完善“三桥十路”和“1212工程”基础上，实施旅游配套、特色打造、环境提升、路桥建设、交通通达、住房改造等城市建设6大工程。重点推进龙门大桥、西二环路、万福东路等建设，打通市区西部和东部快速环线，拉大城市框架；继续实施阳江南路、阳江北路、新建路、芳香路等道路建设，开工建设中隐路二期机场路至南溪河段、中隐路至西二环、琴潭南路、湖塘路，完善老城区路网；继续完善供水管网、天然气管网及加压站、城市光纤等市政基础设施。加快启用城北桂林汽车站，规划建设城南、城东汽车站，积极推进城北公交站、始发站换乘体系等公交枢纽场站及市区停车场库建设。启动旅游、文化、休闲、商业广场等一批特色街区建设。强力推进秀峰区琴潭旅游文化休闲园、叠彩区商贸物流园、象山区旅游度假园、高新(七星)区产业园、雁山区科教园等特色园区建设。加快“两江四湖”二期工程、漓江城市段截污及河堤岸线生态改造工程建设，提升改造漓江等环城水系、机场路、桂磨路两侧区域。推进旧城片区、老村改造以及建筑第五立面改造。启动城中村改造试点。实施县城、重点镇城镇面貌改观“三年行动计划”[22]，“百镇千村行动计划”[23]，着力打造一批特色工贸、特色旅游、特色文化、特色生态村镇。

提高城市管理水平。健全“两级政府、三级管理、四级网络”城市管理体制，实施城市管理综合执法。规范城市道路和公共场地临时停车管理，提升道路通畅能力，缓解城市交通压力。推行数字化城市管理，建立市级数字城管网络。加强市场和社区管理，加大对漓江流域城市段两岸乱搭乱盖的综合整治力度。加强对城乡结合部、城中村、小街小巷、江河湖塘沿岸、交通要道、农贸市场和建筑工地周边区域的市容卫生整治，及时制止违法建设和乱搭乱盖行为。

（六）以提升软实力为目标，推动文化建设新进步

继续实施文化建设五大工程[24]，推进文化事业发展，加强文化基础建设，推动文化产业振兴，创建全国文明城市。

繁荣发展文化事业。实施文化精神工程，把社会主义核心价值体系建设融入文化建设的全过程，积极开展精神文明创建活动，提高市民文明素质。实施文化精品工程，弘扬传统文化，打造新编历史桂剧《灵渠长歌》等一批艺术精品，积极参与中宣部第十二届“五个一工程”评奖活动，继续办好“漓江之声”“百姓大舞台”“百姓大讲坛”“读书月”等品牌文化活动，鼓励各县(区)开展特色节事文化活动，促进县(区)经济发展。实施文化管理工程，加快经营性文化出版印刷事业单位改制步伐，积极培育文化市场，加强“扫黄打非”工作，切实保障文化市场秩序良好。

加强文化基础建设。实施文化景观工程，继续加快“一院两馆”等标志性文化项目建设，推进八路军桂林办事处纪念馆综合楼改扩建、抗战历史文化一条街等项目建设。充分利用漓江剧院、省立艺术馆等演艺场所，推进文化演艺事业发展。加大广西文场、桂剧、彩调等非物质文化遗产的保护、传承和展示力度。实施文化惠民工程，积极推进社区文化中心、乡镇综合文化站、村级文化室、文化信息资源共享、农家书屋建设，推进广播电视“村村通”和农村电影数字化放映，新建24个乡镇综合文化站和91个村级公共服务中心。积极培养各类文化人才。加强综合档案馆舍建设。

加快文化产业发展。实施文化产业发展规划，以培育发展文化休闲、文化演艺、艺术品、传媒影视、出版发行、动漫和网络游戏等产业为重点，努力构建特色文化产业体系。培育一批有实力的骨干文化企业，将文化资源优势转化为产业优势，继续加大对“印象·刘三姐”“愚自乐园”和临桂五通“农民画”等国家文化产业示范基地支持力度。推动高新区创意产业园项目建设，努力将高新区打造成全区动漫培训、认证、交易基地和全国动漫产业基地。

（七）以科学保护漓江为重点，提升可持续发展新水平

严格保护漓江生态，强化节能减排，发展循环经济，努力建设资源节约型和环境友好型社会。

加强漓江生态保护。积极争取《漓江风景名胜区总体规划》和《广西壮族自治区桂林漓江生态环境保护条例》出台，加快构建科学保护漓江长效机制，继续实施漓江源头水源林保护、两岸绿化美化、两岸及水域环境保护、两岸富民惠民、水域管理和景区旅游产品策划等六大工程，全力抓好桂林市防洪及漓江补水枢纽工程和漓江小流域综合治理工程，加快漓江流域乡镇农村环境设施建设。探索建立漓江流域生态补偿机制，积极争取把“漓江保护”列为国家生态补偿财政转移支付基金试点，实施生态补偿措施，使沿江群众在科学保护漓江中受益。

强化节能减排工作。认真落实节能减排责任制、问责制和“一票否决制”，市本级财政继续安排不低于2000万专项资金，重点支持城市污水处理管网系统完善、工业园(集中)区污水处理设施、路灯节能改造等节能减排项目建设。完成各县垃圾处理项目建设，苏桥、秧塘工业园污水集中处理设施力争在年底前建成并投入使用。把年耗能5000吨标准煤以上企业纳入重点监督对象，对超耗超排企业实行挂牌督办，淘汰落后生产能力和污染严重企业。万元GDP能耗及二氧化碳、二氧化硫、化学需氧量、氨氮和氮氧化物减排达到自治区标准，公共机构水、电、油、材等消耗指标下降5%。

推进城乡生态建设。扎实推进“绿满八桂”造林绿化工程和漓江沿岸“四化”工程，完成造林绿化47万亩。推进漓东公园、黑山植物园二期工程建设，加大洲岛、学校、社区和机关等公共绿地建设力度。加强农村生态能源建设，重点发展大中型沼气池和沼气原料发酵新技术。严格实行耕地保护目标责任制，加强水土保持和地质灾害防治。大力推进生态县、生态乡镇、生态村建设。继续开展城市环境综合整治，推进“城乡清洁工程”规范化。争创国家生态园林城市，申办第二届广西园林博览会。

（八）以改革创新为动力，构筑开放合作新格局

以更大决心和勇气推进改革，加快构建充满活力、富有效率、有利于科学发展的体制机制，以改革促创新、以创新促发展。

推进重点领域改革。在重点推进两大综合改革基础上，努力在重要领域和关键环节上取得突破。理顺临桂新区体制机制，抓好服务业、旅游业管理体制机制改革。加快推进城乡统筹改革试点、扩权强镇试点和扩权强县改革。稳步推进政府机构改革、政府采购改革和城市路桥收费改革，继续深化国有企业资产重组、文化教育、医药卫生、华侨农场、供销社等方面改革。抓好农村土地承包和流转规范管理工作，促进农村土地适度规模经营。全面完成集体林权制度主体改革，完善和落实征地、搬迁农民权益保护政策措施。

提高开放合作水平。全方位、多层次、宽领域扩大开放，主动融入“两区一带”区域发展，积极参与区域合作。拓展对台经贸合作领域，加强与欧美、日韩、东盟、港澳台等国家和地区经济文化交流，积极参与第八届中国—东盟博览会。着力破解园区土地、优惠政策、服务环境等招商难题，深化与大集团、大企业合作对接，继续推进产业招商、专题招商和园区招商，力争全年引进项目400个以上、资金388亿元以上，实际利用外资2.75亿美元以上，招商引资实际到位资金增长25%。开展创建大兑现工作示范县和示范园区活动，提高项目履约率、开竣工率、资金到位率。大力实施“走出去”战略，不断优化出口产品结构，开拓外贸新兴市场。

提高科技创新能力。启动实施新一轮科技创新计划，继续推进科技“355工程”，参与国家重大科技项目和自治区千亿元产业重大科技项目攻关，加强国家科技兴贸创新基地(生物医药)和自治区科技兴贸创新基地(橡胶类)建设。加大国家重点实验室、国家级工程技术研究中心和企业技术中心的培育，构建和完善区域创新体系。推进科技成果转化，加强知识产权保护。实施民生科技行动，开展以“科技进企业”“科技进社区”“科技下乡”为重点的科普活动。

（九）以激活民间资本为抓手，引领民营经济新腾飞

全面促进全民创业，放手发展民营经济，推动民营经济加快发展，力争民营经济增加值增长20%以上。

增加民间资本投入。按照非禁即准的原则，最大限度消除民间投资限制。以空间换时间、以资源换产业、以存量换增量，支持民营企业参与国有和集体企业的改制重组。制定鼓励民营企业投资名录，筛选充实一批鼓励和支持类项目，进一步扩大项目建设向民间资本开放。

大力支持民营企业。贯彻落实促进民营经济发展的政策措施，从财税、金融、担保、土地等方面加大对民营经济支持力度。选择5家符合国家产业政策、具有高成长性的民营企业，进行上市融资培育工作。支持民营企业产品和服务进入政府采购目录。实施民营企业人才培养工程，大力支持民营企业技术改造和发展自主品牌。

大力促进全民创业。采取“投资者出一点、财政补一点、金融机构贷一点”运作模式，大力发展创业成本低、成果见效快的微型企业。健全完善创业服务指导中心，加快创业园、创业“孵化”基地建设步伐，进一步激活创业主体，大力实施“回乡创业”工程，争取更多人回乡创办企业。

（十）以保障和改善民生为根本，开创和谐社会建设新局面

坚持服务于民、谋利于民、造福于民，完善基本公共服务体系，着力解决人民群众最关心、最直接、最现实的利益问题。

加强就业和社会保障。实施职业培训计划，建立就业援助机制，新增城镇就业6万人以上，新增农村劳动力转移就业8.50万人以上。建立健全覆盖城乡的社会保障体系，努力实现应保尽保，实现企业在职职工基本养老、失业、工伤和生育保险参保人数分别达36.50万人、23.50万人、30万人、26万人。稳步推进城镇居民基本医疗保险门诊统筹，做好第二批新农保试点工作。落实五保供养、优抚、灾害救济等政策规定，完善社会救助体系，发展社会福利和慈善事业。加大保障性安居工程建设和农村危房改造力度，逐步解决城乡低收入家庭住房困难问题。

全面发展社会事业。组织实施教育发展“十项重点工程”[25]“十项改革试点”[26]，启动灵川、龙胜两县全区学前教育试点工作。加强中等职业教育，进一步提升普通高中内涵建设。加快桂林市职业教育中心学校临桂分校、桂林中学临桂校区等项目建设；继续推进中小学校舍安全工程，确保教育系统安全和谐稳定。大力支持驻桂高校建设，做大做强国际旅游教育基地。健全城乡基层医疗卫生服务体系，加强城市医院急重症救治能力及新区医疗服务能力建设，有效应对各类卫生突发事件。继续抓好创建全国无障碍城市、全国人口和计划生育综合改革示范市工作，扎实推进“诚信计生”。广泛开展全民健身活动，积极组织参加自治区第十二届运动会，争取获得优异成绩。办好龙胜各族自治县60周年县庆。

全力维护社会稳定。继续推进政法三项重点工作[27]，深入开展和谐建设在基层活动，健全信访工作责任制和调解机制，提高处置突发公共事件能力，妥善调处各类社会矛盾。启动“六五”普法工作，加强律师队伍建设。推进“天网”工程建设，加大社会治安综合治理力度，严厉打击各类刑事犯罪。开展安全生产基层基础年活动，实施质量兴市战略，强化食品、药品监督管理。扎实做好国防动员、民兵预备役、人民防空和双拥工作，继续争创“全国双拥模范城”。

深入实施惠民工程。继续落实国家各项惠民政策，筹措资金30亿元以上，做好10项惠民工程：

1.医疗卫生保障惠民工程。实施城镇职工、城镇居民基本医疗保险和新型农村合作医疗保险，3项参保率均达90%以上；提高参合农民、参保居民的年人均财政补助标准；继续实施基本公共卫生服务项目，免费为城乡居民提供建立居民健康档案、健康教育、预防接种等9类基本公共卫生服务；实施艾滋病防治攻坚工程；建设标准化村级卫生室;完成农村改厕1.3万座。

2.社会保障惠民工程。完成城镇企业职工基本养老保险新增参保任务；做好平乐县、恭城瑶族自治县农村新型社会养老保险试点工作；按照自治区要求提高城乡低保对象补助标准和新建农村五保村；继续实施“阳光家园计划”项目，为3000名残疾人提供日间照料和居家托养服务。

3.安居惠民工程。完成城镇保障性安居改造和农村危房改造任务；开工建设廉租房1812套，实现交付使用1000套目标；开工建设经济适用房27.27万平方米，竣工25.32万平方米。

4.教育惠民工程。对各县(区)中小学校舍进行新建、迁建和安全加固；对就读普通高中的库区移民子女和在国家级贫困县就读的普通高中生免学费；对农村义务教育阶段家庭经济困难寄宿生给予生活费补助；对农村义务教育学校课桌椅进行更新改造；对中等职业教育学生给予资助；对困难残障就读学生给予资助；对考上大学的贫困新生给予路费和短期生活费资助；改善少数民族和民族地区中小学校基础设施建设。

5.文化惠农工程。完成91个村级公共服务中心建设、每个行政村每月放映1场电影、实施20户以下自然村(屯)通广播电视工程。

6.生态惠民工程。完成通道绿化和村(屯)绿化等项目建设任务；对1316万亩国家级和自治区级公益林进行生态效益补偿；新建户用沼气池1万座。

7.强农惠农工程。实施农村“一事一议”奖补项目；继续做好“万村千乡”市场工程，建设配送中心5家、农家店270家；实施“菜篮子工程”，重点建设灵川、临桂、雁山菜篮子生产基地。

8.强基惠农工程。完成土地整理项目建设；支持农业综合开发土地治理项目建设；继续实施1500个自然村(屯)内道路硬化、30个行政村通油路或水泥路项目；继续实施农村安全饮水工程，解决20万农村人口的饮水安全问题。

9.新村建设惠民工程。实施“普惠制”新村建设项目，完成400个村建设任务；做好水库移民新村建设工程。

10.城市环境治理惠民工程。继续实施“两江四湖”二期(桃花江)、南溪河、小东江环境综合整治工程；对市区部分街区路灯进行节能改造；开工建设漓江(市区段)截污工程、灵剑溪环境综合整治工程；启动城北水厂二期扩建工程前期工作。

各位代表，实现“十二五”的宏伟蓝图，完成今年的工作目标任务，对政府建设提出了更高的要求，我们要增强贯彻落实科学发展观和加快转变经济发展方式的自觉性、坚定性，努力建设人民满意的政府！一是进一步转变政府职能，建设高效政府。着力提高公务员队伍综合素质，加强执行能力建设，认真落实首问首办责任制、限时办结和责任追究制，提高政府行政效能。二是进一步转变工作作风，建设服务政府。坚持把群众根本利益作为政府工作的出发点和落脚点，戒空、戒虚、戒假、戒骄、戒懒、戒奢，注重服务基层，服务企业，努力解决关系群众切身利益的突出问题。三是进一步加强依法行政，建设法治政府。自觉接受人大及其常委会的法律监督、工作监督和政协民主监督，建立完善政府部门与民主党派、工商联对口联系制度，严格、公正、文明执法，切实提高依法行政水平。四是进一步加强廉政工作，建设廉洁政府。完善惩治和预防腐败体系，做到勤勉尽责、廉政务实，努力做出经得起人民和历史检验的实绩！

各位代表！桂林未来五年的发展蓝图已经绘就，新的形势催人奋进，新的征程任重道远。抚今追昔，我们信心百倍；鉴往知来，我们豪情满怀。让我们紧密地团结在以胡锦涛同志为总书记的党中央周围，高举中国特色社会主义伟大旗帜，深入贯彻落实科学发展观，在市委的领导下，以更加昂扬的斗志、更加进取的精神、更加务实的作风，团结一致、埋头苦干、奋发图强，扎实推进工业化、城镇化、农业产业化，为实现“十二五”规划目标，为实现“富民强市”新跨越而努力奋斗！
